# Folk use of medicinal plants in Karst and Gorjanci, Slovenia

**DOI:** 10.1186/s13002-017-0144-0

**Published:** 2017-02-23

**Authors:** Mateja Lumpert, Samo Kreft

**Affiliations:** Faculty of Pharmacy, University of Ljubljana, Aškerčeva cesta 7, SI-1000 Ljubljana, Slovenia

**Keywords:** Ethnomedicine, Folk knowledge, Ethnobotany, Balkan, Comparison, Herbal preparations

## Abstract

**Background:**

Information on the use of medicinal plants in Karst and Gorjanci is not available in the literature, but collection of plants is still an important and widespread practice in these regions. Karst and Gorjanci are two remote regions in Slovenia that are only 120 km apart but have different climates; one region is close to the Italian border, and the other is near the Croatian border. Our aim was to report and compare the use of medicinal plants in both regions.

**Methods:**

From October 2013 to September 2014, 25 informants each in Karst and Gorjanci were interviewed during field research. The age of the informants ranged from 33 to 89 years, with an average age of 61 years in Karst and 69 years in Gorjanci. The main question was “Which plants do or did you collect from nature and use?” Plants of medicinal, nutritive, veterinary or cosmetic use were considered.

**Results:**

A total of 78 and 82 taxa were reported in Karst and Gorjanci, respectively; 65 taxa were reported in both regions. Approximately 64% of the plants in each region were distinctive for only a few informants (fewer than 7). The remaining plants were considered important, and the majority were mutual to both regions. Few reported plants were typical for just one region. Differences in the use of some common medicinal plants were observed, e.g., *Matricaria chamomilla* was used mostly for the treatment of gastrointestinal disorders, respiratory infections and sore eyes in Gorjanci but as a calmative in Karst. Altogether, 15 different oral and 15 different topical medicinal preparations were reported. Folk knowledge was found to be influenced by the media, particularly popular books about medicinal plants that were published in the 20th century.

**Conclusions:**

The present research documents the folk use of medicinal plants in Karst and Gorjanci, Slovenia. This rapidly changing practice needs to be documented before it disappears or changes.

## Background

In Slovenia, knowledge about plants is transmitted from generation to generation and is also influenced by written sources. The beginning of this practice goes back to *Comments of Dioscorides* written by Pietro Andrea Mattioli. He lived and worked from 1540 to 1554 in Gorica, a town in northeastern Italy populated by a Slovene-speaking minority, and he was the first to describe plants of Slovenian flora [1, 2]. In the 18th and 19th centuries, folk healers in Slovenian ethnic territory used folk medicine manuscripts, which were translations of mostly German medicine and veterinary books, especially herbals (or Kräuterbücher) from the 16th century and the beginning of the 17th century [1, 3] Most manuscripts were translations of *Gart der Gesundheit* (1485), *Kreutterbuch* by Pietro Andrea Mattioli (1590), *Neu Vollkommenes Kräuter-Buch* by Pietro Andrea Mattioli (1678), *Vollständiges Kräuterbuch* by Adam Lonicer (1557), and *Neu Vollkomentlich Kreuterbuch* by Jakob Tabernaemontanus (1613). Later, the translated books were manually transcribed many times, and the transcribers added their own observations to the manuscripts [1]. In the second half of the 19th century, the first original (non-translated) Slovenian works about medicinal plants were published [4, 5], and manuals for the wild collection, drying and use of Slovenian medicinal plants were issued later [6–9]. Throughout the 20th century, there was steady growth of published books about medicinal plants; some of them were original Slovenian works, and some were translations from foreign authors; most were written by pharmacists and only some by folk healers [10–27].

The use of plants has been scarcely investigated in Slovenia, and only a few ethnobotanical studies have been conducted. The Karst region is a limestone plateau in southwestern Slovenia that continues on the Italian side of the border [28]. The Italian part of the Karst region, also known as Trieste Karst, was included in an ethnobotanical study of the Venezia Giulia region in 1988, and a list of 59 plants that were used in Trieste Karst was reported [29]. Guštin Grilanc investigated the folk herbalist tradition in both the Italian and Slovenian parts of the Karst region and published a list of 124 plants used for healing, food, toys, superstitions, and folk traditions with short descriptions [30]; however, the methodology of the work was not described, and only a detailed list of informants was given. Gorjanci is a mountain range in southeastern Slovenia that runs southwest to northeast along the Croatian border [31]. From 1950 to 1983, ethnographic researchers collected testimonials on the natural and magical treatment of the people in Dolenjska and Bela Krajina, two regions where Gorjanci is located. Makarovič [32] analyzed the collected testimonials and concluded that the ethnographers’ notes contained random and generalized data on knowledge about natural medicines and magical practices; those data were collected unsystematically and were incomplete. As a result, this analysis provided a very rough estimation of the use of medicinal plants. A total of 112 plants were mentioned (botanical names were often missing, and only the local names were given), and the most frequent were *Matricaria chamomilla*, *Sambucus nigra*, *Allium sativum*, *Juniperus communis*, *Tilia platyphyllos*, *Allium cepa*, and *Sempervivum tectorum*.

In addition, some socio-economic studies [33–35] investigated the plants people use in Slovenia. According to these studies, the most frequently used plants in Slovenia were *Achillea millefolium*, *Hypericum perforatum*, *Matricaria chamomilla*, *Mentha piperita*, *Salvia officinalis*, *Sambucus nigra*, *Tilia platyphyllos*, and *Urtica dioica* (Table 1). A survey on wild-growing edible plants and human nutrition was conducted using a Slovenian cookbook and informants from different regions of Slovenia. According to the informants, the five most frequently reported taxa were *Taraxacum officinale* agg., *Fragaria* sp., *Castanea sativa*, *Vaccinium myrtillus* and *Sambucus nigra*; according to the cookbooks, the five most frequently mentioned taxa were *Juglans regia*, *Armoracia rusticana*, *Castanea sativa*, *Corylus avellana*, and *Taraxacum officinale* agg. [36].

**Table 1:** Most frequently used plants in Slovenia, as reported in previous socio-economic studies [33–35]

Plant species	References			
	Plants for infusions [9]	Plants in herbal medicinal products [9]	Plants and plant preparations [10]	Plants for abdominal pain, headache, diarrhea, and fever [11]
*Achillea millefolium*	X	X	X	X
*Arctostaphylos uva-ursi*	X			
*Arnica montana*			X	
*Calendula officinalis*		X		
*Centaurium minus*				X
*Echinacea purpurea*		X		
*Hypericum perforatum*		X	X	X
*Matricaria chamomilla*	X		X	X
*Mentha piperita*	X		X	X
*Rosa canina*				X
*Salvia officinalis*	X	X	X	X
*Sambucus nigra*	X	X	X	X
*Tilia platyphyllos*	X	X	X	X
*Urtica dioica*	X	X	X	
*Vaccinium myrtillus*				X
*Valeriana officinalis*		X		

Similar to the neighboring countries of Austria [37, 38] and Italy [39–42], the wild collection of plants is also important in Slovenia. According to a survey conducted in seven pharmacies [34], the informants obtained medicinal plants from a pharmacy (68%) or by wild collection (48%). Other possible sources were friends and relatives (33%), specialized shops (19%), and herbalists (17%). In another study conducted in the city of Velenje, the informants obtained medicinal plants by wild collection (37%), from relatives (25%), from a pharmacy (24%) or from a market (5%) [35].

Limited information about the folk use of plants for medicinal and nutritive purposes is available for Slovenia. This paper reports the results of a study on the use of plants in two areas in Slovenia: Gorjanci in southeastern Slovenia and Karst in western Slovenia. The areas are approximately 120 km apart; one is close to the Italian border, and the other is close to the Croatian border. Except for one mountain pass, Gorjanci is rather impassable to Croatia, whereas Karst is more passable to Italy and is the hinterland of Trieste, which has been a trading port for centuries. Karst is a transitional region with Mediterranean and continental influences, and Gorjanci has a moderate continental climate. Both areas are rural and remote, and the landscape is only partially cultivated. They are inhabited by a Slovene population (approximately 95%). In the past, the inhabitants were farmers, but the active population currently consists of daily migrants to industrial centers. People are still connected to nature, and knowledge about plants is important in their lives because they produce their own food in gardens or fields and some are still farmers [28, 31]. This study focused on plants for medicinal use but also includes plants for nutritive, cosmetic and veterinary uses. This study aimed to investigate the following: 1) the plants used in villages in the foothills of Gorjanci and in Karst, 2) the plant preparations and purposes for their use, and 3) the differences in the use of plants between Gorjanci and Karst; these could be the result of differences in climate, vegetation or connections to neighboring areas.

## Methods

### Research area

**Fig. 1:**
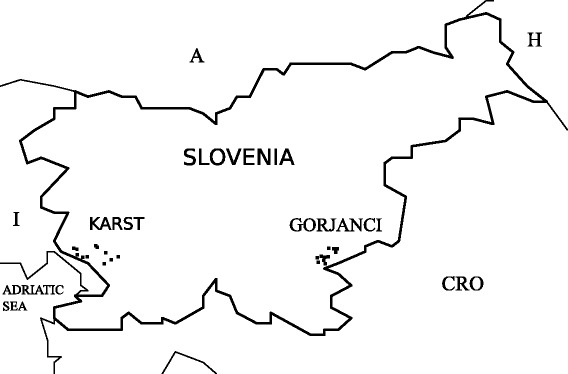
Map of the study area. The villages where the interviews were conducted are represented by *dots*

The use of plants was investigated through interviews with local people in villages in the Karst plateau in southwestern Slovenia and in villages in the foothills of the Gorjanci mountain range in southeastern Slovenia (Fig. 1). The interviews were conducted from October to December 2013 and from May to September 2014. The villages in Karst included Pliskovica, Veliki dol, Brje pri Komnu, Gorjansko, Klanec pri Komnu, Štanjel, Kobdilj, Grahovo Brdo, Griže and Štjak (altitudes ranging from 184 to 518 m above sea level). The villages in Gorjanci included Iglenik, Vrhe, Dolž, Mali Cerovec, Pangrč Grm, Sela pri Zajčjem Vrhu, Stopiče, Dolenji Suhadol, Cerov Log and Mihovo (altitudes ranging from 232 to 442 m above sea level).

Karst is a limestone plateau in southwestern Slovenia. The climate has Mediterranean and continental influences [28], and the mean precipitation ranges from 1400 to 1650 mm per year [43]. The mean January and July temperatures are 2.4 and 20.8 °C, respectively. The predominant bedrock is limestone, which is vulnerable to corrosion. The bedrock is covered with chromic cambisol, chromic luvisol and rendzina. The natural forests are composed of pubescent oak (*Quercus pubescens*), hop hornbeam (*Ostrya carpinifolia*) and flowering ash (*Fraxinus ornus*) [44]. In the 19th century, parts of Karst were afforested with black pine (*Pinus nigra*). The population density is 41 people per km^2^, and the farming population represented 4.1% of the total population in 1991. There are 100 settlements [28]. Industrialization with employment began after the Second World War and advanced after 1960. The active population began to travel daily from rural areas to the surrounding industrial centers [45].

Gorjanci is a mountain range in southeastern Slovenia. It has a moderate continental climate with mean precipitation between 1100 and 1300 mm per year. In the foothills, the mean January and July temperatures are approximately 0 and 20 °C, respectively. Gorjanci consists primarily of limestone and dolomite and is covered with rendzina and chromic cambisol. The higher regions of Gorjanci are overgrown with extensive beech forests (*Fagus sylvatica*). Some areas have been deforested for pasture and are now becoming naturally overgrown or reforested with spruce (*Picea abies*) and fir (*Abies alba*). Forests of beech (*Fagus sylvatica*) and hop hornbeam (*Ostrya carpinifolia*) grow in the eastern part of Gorjanci. Oak (*Quercus petraea*) forests grow in the western and central parts of Gorjanci up to 400 and 600 m in altitude, respectively. The area is sparsely populated; the population density was 27 people per km^2^ in 1991. The highest number of inhabitants, 8000, occurred at the beginning of World War II. This remote area is characterized by the depopulation of young people, who migrate to cities. There are 87 settlements, most of which are located on the periphery. In 1961, two-thirds of the population were farmers, whereas 22.2% of the population was farmers in 1991. Farmers produce livestock and mostly grow fodder plants [31].

### Data collection

In the study, 25 informants each were interviewed in Karst and in Gorjanci. The age of the informants ranged from 33 to 89 years, with an arithmetic mean age of 61 years in Karst and 69 years in Gorjanci (Table 2). In Karst, 23 informants were originally from the Karst region, and two informants were born near that region. In Gorjanci, 17 informants were originally from villages in the foothills of Gorjanci, and 8 informants were born close to that region (30 km radius). Purposive and snowball sampling methods were used in this study [46, 47]. Informants were contacted in villages (on the street, outside their house or in the field). They were often recommended by other members of their family as being more knowledgeable and by other informants in their village or neighboring villages. The informants were recruited without any selection based on sex, age or social status. The interviews were performed in the homes of the informants and in the fields. The language used in the interviews was Slovenian. The informants were made aware of the scope of the study, and informed consent was obtained.

**Table 2:** Informants’ age and sex in Karst and Gorjanci

	Average age (years)	Age (years)			Sex	
		33–49	50–69	70–89	Male	Female
Karst	61	4	15	6	6	19
Gorjanci	69	5	4	16	1	24

### Interviews and statistical analysis

Free-listing and semi-structured interviews were used to collect data [47, 48]. At the beginning of the interview, the informants were asked to list the plants that they use currently or used in the past and that are collected from nature. They were asked the following free-listing question: “Which plants do or did you collect from nature and use?” Then, they were asked the following questions for each plant:

- *Which part of the plant do you use?*
- *How do you prepare it?*
- *What is it good for?*
- *Who would you recommend it to?*

At the end of the interview, the informants were also asked to answer the following questions about the sources of their knowledge:

- *“Where did you get your knowledge about plants?” and “Did anybody show or tell you something about plants?”*

The informants’ answers were written in a notebook and then entered and organized in worksheets in Microsoft Excel 2010 [49]. Statistical analyses were performed using MS Excel, Visual Anthropac [50], and SPSS [51]. Visual Anthropac was used to perform an analysis of the free-list data in which frequency, rank, and Smith’s salience index were calculated. Rank (order) and frequency of mention are two measures of importance (salience) that are combined into a single index called the Smith’s salience index [52]. Other statistical analyses were performed using MS Excel and SPSS. SPSS was used to perform Pearson’s chi-squared test and Fisher’s exact test. Since purposive sampling was used for informant selection, the results of the statistical analysis are limited to the population under study [46].

### Plant material

Plant samples (fresh plants, dried plants and sometimes herbal preparations) were collected at informants’ homes or nearby when possible (sometimes this was not possible due to the condition of the informant’s health or the weather). When possible, more knowledgeable informants were followed into the field to show us the reported plants. The plants were identified by both authors and stored at the herbarium of the Faculty of Pharmacy, University of Ljubljana. Cultivated plants were not collected. The plant nomenclature followed *Mala flora Slovenije* [53] and was checked using the online database The Plant List (http://www.theplantlist.org/). In some cases, which are reported in the results section, the informants used several species belonging to the same genus for the same purposes, although they were aware that they were different species (*Artemisia* spp., *Mentha* spp., *Plantago* spp., *Quercus* spp., and *Trifolium* spp.). Furthermore, in some cases, the informants did not distinguish among different species belonging to the same genus (*Asparagus* spp., *Crataegus* spp., *Centaurium* spp., *Lavandula* spp., *Rosa* spp., *Rubus* spp., *Solidago* spp., and *Tilia* spp.).

## Results and discussion

### General data on the plants reported in Karst and Gorjanci

In each region, 25 informants were interviewed and reported a total of 94 plants, including 77 in Karst and 81 in Gorjanci; 64 plants were reported in both regions (Table 3).

**Table 3:** Medicinal (MED), veterinary (VET), nutritional (NUT), cosmetic (COS) and other (OTHER) uses of wild (W) and cultivated (C) plants in Karst and Gorjanci. The data were gathered from interviews with 25 informants in Karst and 25 informants in Gorjanci

No. of informants in Karst	No. of informants in Gorjanci	Botanical taxon and family	Slovenian name	Status	Part(s) used	Preparation and administration	Recorded uses in Karst	Recorded uses in Gorjanci
14	20	*Achillea millefolium* L. (Asteraceae)	Rman	W	Flower	Infusion, oral application	MED: treatment of stomach and digestive problems, menstrual cramps and gynecological problems	MED: treatment of stomach and digestive problems, menstrual cramps, women’s problems; to cleanse the body and kidneys; diuretic; treatment of cold NUT
						Fresh flowers are wrapped around the hand	×	MED: to heal sore hands
						Browned flour soup with yarrow flowers (soup made by roasting and browning flour, roasting yarrow flowers and adding water and two eggs)	×	MED: treatment of intestinal problems
						Egg omelet with yarrow flowers	MED: treatment of digestive problems	×
						Ointment made with lard	MED: treatment of hemorrhoids	×
						Herbal liqueur	×	NUT/MED
3	2	*Aesculus hippocastanum* L. (Hippocastanaceae)	Divji kostanj	C	Fruit	Maceration in schnapps, topical application	MED: treatment of varicose veins	MED: treatment of spinal inflammation
						Chestnuts are placed in a bag, and the bag is rubbed on the feet	MED: treatment of pain in the legs	MED: to promote better blood circulation in legs
					Flower	Maceration in schnapps, topical application	MED: treatment of varicose veins	×
						Herbal liqueur	×	NUT/MED
2	2	*Allium cepa* L. (Alliaceae)	Čebula	C	Bulb	Fried pieces of onion are placed on the skin	×	MED: to heal furuncles and abscesses
						Onions and honey, oral application	MED: treatment of bronchitis and sore throat	×
1	1	*Allium sativum L.* (Alliaceae)	Česen	C	Clove	Necklace made of garlic cloves is worn around the neck	×	MED: anthelmintic
						Tincture	Unknown use	×
2	4	*Allium ursinum* L. (Alliaceae)	Čemaž	W	Leaf	Salad, spread made with olive oil	NUT	NUT: good for cleansing, purifier of vessels, to decrease blood pressure (the informants did not eat *A. ursinum* for medicinal purposes)
						Maceration in schnapps	×	NUT: good for everything
5	0	*Aloysia citrodora* Palau (Verbenaceae)	Leviš, citronka	C	Leaf	Infusion	NUT	×
						Maceration in schnapps with sugar	NUT	×
6	2	*Althaea officinalis* L. (Malvaceae)	Slez, ajbiš	C	Leaf	Infusion, oral application	×	MED: treatment of cough, good for the respiratory system
						Fresh leaf is placed on a wound	MED: to heal wounds	×
						Scalded leaves are placed on damaged skin (skin punctured with a nail or thorn that becomes infected, called “pasjica” in Karst)	MED: to heal wounds, to pull pus out of the wound	×
						Leaves cooked in milk, oral application	MED: treatment of sore throat	×
						Herbal liqueur	NUT/MED	NUT/MED
4	2	*Anthyllis vulneraria* L. (Fabaceae)	Ranjak	W	Flower	Infusion, topical application	×	MED: to rinse and heal wounds
						Infusion, oral application	NUT	×
						Herbal liqueur	×	NUT/MED
1	0	*Armoracia rusticana* G. Gaertn., B. Mey. & Schreb. (Brassicaceae)	Hren	W/C	Root	Grated root eaten with other Easter dishes	NUT	×
6	12	*Arnica montana* L. (Asteraceae)	Arnika	W	Flower	Maceration in olive oil, topical application	MED: to heal wounds, burns, scabs and contusions	MED: to massage painful areas, rheumatism
						Maceration in schnapps, topical application	MED: to massage painful areas (knees)	MED: to disinfect wounds, to heal wounds, treatment of inflamed wounds, rashes, burns and stings
						Maceration in schnapps, oral application	×	MED: to heal stomach problems
						Herbal liqueur	NUT/MED	NUT/MED
7	6	*Artemisia* (*Artemisia vulgaris* L., *Artemisia absinthium* L.) (Asteraceae)	Pelin	W/C	Aerial parts (of young plants) and leaves	Wormwood is placed in wine for a short time, for drinking (usually with fatty food)	×	MED: to promote appetite and digestion, good for the stomach NUT: a drink with fatty foods, invigoration in spring
						Infusion, oral application	×	MED: treatment of stomach and digestive problems
						Infusion for spraying vines		OTHER: to prevent or treat vine diseases
						Maceration in schnapps, oral application	MED: treatment of digestive problems, good for the stomach	MED: treatment of stomach problems
						Wormwood is placed in water for a short time for drinking	NUT: to quench thirst	×
						Whole plant	VET: at the loss of appetite	×
						Herbal liqueur	NUT/MED	NUT/MED
6	0	*Asparagus* spp. (*Asparagus acutifolius* L., *Asparagus tenuifolius* Lam.) (Asparagaceae)	Šparglji	W	Young shoots	Prepared in different dishes (egg omelet, soup, minestrone soup)	NUT: blood cleansing, diuretic, believed to be good for the kidneys (the informants did not eat asparagus for medicinal purposes)	×
1	4	*Bellis perennis* L. (Asteraceae)	Marjetica	W	Flower	Infusion, oral application	MED: good for ill children	MED: treatment of cold and cough
						Infusion for bath	×	COS: for legs
						Herbal liqueur	NUT/MED	NUT/MED
0	3	*Berberis vulgaris* L. (Berberidaceae)	Češmin, češminc	W/C	Fruit	Infusion, oral application	×	MED: antipyretic
0	2	*Beta vulgaris* var. *conditiva* L. (Chenopodiaceae)	Rdeča pesa	C	Root	Juice, oral application	×	MED: antipyretic
1	10	*Betula pendula* Roth (Betulaceae)	Breza	W	Leaf	Infusion, oral application	×	MED: to heal bladder and kidney inflammation, diuretic, to cleanse blood and body, antidiabetic
						Infusion for bath	×	MED: to prevent sweaty feet
						Leaves are wrapped around the legs	×	MED: treatment of leg pain
						Maceration in vinegar, topical application	COS: good for the scalp	×
1	1	*Brassica oleracea* var. *capitata* L. (Brassicaceae)	Glavnato zelje, zelje	C	Leaf	Fresh leaves are wrapped around the knee or placed on the back	MED: treatment of back pain	MED: to pull the water out of the knee
9	12	*Calendula officinalis* L. (Asteraceae)	Ognjič	C	Flower	Infusion, oral application	MED: good for intestinal problems, against diarrhea NUT	MED: treatment of stomach pain NUT
						Ointment made with lard or fat from the abdomen of a chicken, topical application	MED: to heal burns, contusions, wounds and eczema COS: treatment of dry skin on soles and hands	MED: to heal sunburns, wounds, bruising, and eczema; to massage painful areas (knees, shoulders, joints) COS: treatment of dry skin and hard skin on the heels
						Maceration in oil, topical application	×	MED: to heal burns
						Maceration in schnapps, topical application	×	MED: to heal wounds
						Herbal liqueur	×	NUT/MED
						Dried flowers, spice	×	NUT: spice
0	2	*Cannabis sativa* L. (Cannabaceae)	Konoplja	C	Aerial parts	Preparation was not reported	×	VET: animal feed for pigs
						Fabric	×	OTHER: fabric for sheets, healthier than cotton
3	7	*Carum carvi* L. (Apiaceae)	Kimelj, kumina	W/C	Fruit	Infusion, oral application	MED: treatment of abdominal cramps and stomach problems, carminative, antiflatulent NUT	MED: treatment of digestive problems, treatment of stomach problems, carminative and antiflatulent, antidiarrheal, protects intestines and stomach NUT
						Spice	×	NUT: spice for sausages, bread and the common local pastry “belokranjska pogača”
1	4	*Castanea sativa* Mill. (Fagaceae)	Kostanj	W	Fruit	Cooked or roasted	NUT	NUT
					Flower	Herbal liqueur	NUT/MED	NUT/MED
11	15	*Centaurium* spp. (*Centaurium erythraea* Rafn, *Centaurium pulchellum* (Sw.) Druce) (Gentianaceae)	Tavžentroža	W	Flowers or aerial parts	Infusion, oral application	MED: treatment of stomach problems, digestive problems, menstrual cramps, menstrual problems, anemia	MED: treatment of stomachache, stomach problems, digestive problems, menstrual problems, cold, flu, antipyretic, to calm nerves, to counteract tiredness, to heal wounds on the gum
						Maceration in schnapps, oral application	MED: treatment of digestive problems, loss of appetite, anemia VET: to heal digestive problems	MED: treatment of stomach problems and stomachache
						Grass with centaury, animal feed	VET: animal is healthier if it feeds on grass containing centaury	×
						Herbal liqueur	NUT/MED	NUT/MED
2	2	*Chelidonium majus* L. (Papaveraceae)	Krvavi mlečnik	W	Latex	Latex, topical application	MED: treatment of warts	MED: treatment of warts
8	2	*Cornus mas* L. (Cornaceae)	Dren	W	Fruit	Maceration in schnapps, oral application	NUT	MED: treatment of abdominal cramps NUT
						Jam	NUT	NUT
						Infusion	NUT	NUT
						Syrup	NUT	×
						Raw fruits	NUT	×
					Flower	Infusion	NUT	×
13	6	*Crataegus* spp. (Rosaceae)	Glog	W	Flower and leaf	Infusion, oral application	MED: good for the heart, good for the prevention of epilepsy, good for nerves NUT	MED: good for the heart NUT
					Fruit	Infusion	NUT	NUT
						Raw fruits	NUT	×
2	1	*Echinacea purpurea* (L.) Moench (Asteraceae)	Ameriški slamnik	C	Flower	Maceration in schnapps, oral application	×	MED: immunostimulant
					Root	Maceration in schnapps, oral application	MED: immunostimulant	×
2	10	*Equisetum arvense* L. (Equisetaceae)	Preslica	W	Aerial parts	Infusion, oral application	MED: to cleanse the kidneys and lungs	MED: diuretic, to cleanse kidneys, blood and body, treatment of urinary tract inflammation
						Herbal liqueur	NUT/MED	NUT/MED
2	0	*Ficus carica* L. (Moraceae)	Figa	C	Fruit	Jam	NUT	×
					Latex	Latex, topical application	MED: treatment of warts	×
13	0	*Foeniculum vulgare* Mill. (Apiaceae)	Koromač (in Karst)	W/C	Flower	Dried crushed flowers, spice (known as “golden dust” in Karst)	NUT: spice for fried livers, fried eggs and browned flower soup	×
					Aerial parts of young plants	Egg omelet with young aerial parts of fennel	NUT	×
					Fruit	Infusion	MED: to heal abdominal cramps, counteracts flatulence, infusion for children	×
5	13	*Fragaria vesca* L. (Rosaceae)	Divja jagoda, gozdna jagoda	W	Fruit	Raw berries	NUT	NUT
					Leaf	Infusion	NUT	NUT
						Herbal liqueur	NUT/MED	NUT/MED
0	3	*Galium odoratum* (L.) Scop. (Rubiaceae)	Dišeča perla	W	Aerial parts of flowering plant	Infusion	×	NUT
						Herbal liqueur	×	NUT/MED
3	1	*Gentiana lutea* L. (Gentianaceae)	Košutnik, encijan, lecijan	W	Root	Maceration in schnapps, oral application	MED: to heal stomach problems NUT	MED: good for the stomach
						Infusion, oral application	VET: to heal intestinal problems (livestock)	×
1	0	*Hedera helix* L. (Araliaceae)	Bršljan	W	Leaf and fruit	Maceration in schnapps, topical application	MED: treatment of contusions	×
11	16	*Hypericum perforatum* L. (Hypericaceae)	Šentjanževka, šentjanževe rože	W	Flower	Maceration in oil, topical application	MED: to heal wounds, contusions, burns and sunburn; to massage painful areas (arthritis); treatment of varicose veins, treatment of back pain COS: to promote tanning of the skin in the sun	MED: to heal wounds, scabs, burns and sunburn; treatment of varicose veins; to massage painful areas and inflamed joints (rheumatism) VET: to heal indigestion in hens (when they eat too much sand)
						Maceration in schnapps, topical application	MED: treatment of varicose veins	MED: to massage painful areas and joints (rheumatism), to heal wounds and contusions
						Infusion, oral application	Indication was not recorded, although the infusion is used	MED: treatment of menstrual and stomach problems, calmative, counteracts depression
						Infusion for bath	×	MED: treatment of tired legs
						Herbal liqueur	×	NUT/MED
					Aerial parts	Maceration in schnapps, topical application	×	MED: to disinfect cuts and wounds
2	0	*Inula hirta* L. (Asteraceae)	Astramontana, strupenica, srkodlakavi oman	W	Flower	Infusion, topical application	MED: to heal wounds, especially poisoned wounds VET: to heal snake bites (dog)	×
						Maceration in schnapps, topical application	MED: to heal wounds, especially poisoned wounds VET: to heal snake bites (dog)	×
						Maceration in schnapps, oral application	MED: good for the stomach	×
2	8	*Juglans regia* L. (Juglandaceae)	Oreh	C	Unripe fruit	Maceration in schnapps	NUT	MED: treatment of digestive disorders and stomachache; counteracts diarrhea VET: to heal intestinal problems (livestock) NUT
						Herbal liqueur	NUT/MED	NUT/MED
					Leaf	Water extract made by cooking leaves in water, topical application	×	VET: to protect animals from horseflies/horsefly repellent (steer/bullock)
12	9	*Juniperus communis* L. (Cupressaceae)	Brin	W	Fruit	Alcoholic distillate from fermented juniper berries, oral application	MED: treatment of digestive problems, stomachache, stomach problems and cramps, to promote better digestion NUT	MED: treatment of menstrual and women’s problems, stomach problems and stomachache, treatment of digestive problems NUT: aperitif, drink in the morning
						Alcoholic distillate from fermented juniper berries topical application	×	MED: treatment of swollen legs
						Essential oil from fermented juniper berries, topical application	MED: to massage painful areas and joints (arthritis and rheumatism), to promote better blood circulation (rubefacient), treatment of cramps and colic in babies and children, to heal wounds, to heal dog bites	MED: treatment of intestinal problems
						Essential oil from fermented juniper berries, topical application around the navel, on the temples, under the nose, on the head, used for children	MED: anthelmintic	MED: anthelmintic
						Essential oil from fermented juniper berries, a small amount is placed on sugar and eaten	MED: treatment of stomachache and digestive problems	MED: antipyretic
						Dried berries, spice	NUT: good for the stomach, sauerkraut, jota (soup)	NUT: to promote better digestion
						Maceration in schnapps, oral application	MED: treatment of stomach problems NUT	×
						Infusion, oral application	×	MED: good for the internal organs
						Bread with essential oil from fermented juniper berries, oral application	×	VET: treatment of intestinal problems (steer/bullock)
						Herbal liqueur	NUT/MED	NUT/MED
					Twig	Infusion, oral application	×	MED: treatment of digestive problems
						Syrup	×	MED: treatment of sore throat
9	2	*Laurus nobilis* L. (Lauraceae)	Lovor	C	Leaf	Spice	NUT: spice for sauerkraut and beans, used in dishes to prevent flatulence	NUT
						Syrup, oral application	MED: treatment of cough, antiflatulent	×
7	2	*Lavandula* spp. (Lamiaceae)	Sivka	C	Flower	Dried flowers placed in sachets	OTHER: household moth repellent	OTHER: household moth repellent
						Infusion, oral application	MED: to calm the nerves	×
						Home distillation of essential oil and hydrosol	COS: good for the skin, to cleanse the skin	×
						Spice	NUT: spice for biscuits	×
4	1	*Levisticum officinale* W.D.J. Koch (Apiaceae)	Luštrek	C	Leaf	Maceration in schnapps, oral application	MED: good for the stomach NUT	×
						Spice	NUT	×
						Infusion, oral application	×	MED: diuretic
0	3	*Linum usitatissimum* L. (Linaceae)	Lan	C	Seed	Linseed is placed in the eye	×	MED: treatment of eye infection, if something falls in the eye
					Aerial parts	Fabric	×	OTHER: fabric for sheets, clothes
0	1	*Malus sylvestris* (L.) Mill. (Rosaceae)	Lesnika	W	Fruit	Vinegar	×	NUT
16	19	*Matricaria chamomilla* L. (Asteraceae)	Kamilica	W/C	Flower	Infusion, oral application	MED: digestion, flatulence, abdominal cramps, to calm the stomach, to calm the nerves, to heal sore throat, for women’s problems	MED: treatment of abdominal and menstrual cramps; for digestive, stomach and women’s problems; to counteract nausea, to enable vomiting, to calm the stomach, treatment of cold, flu and cough, to calm babies, infusion for women giving birth NUT
						Infusion, topical application	MED: to rinse sore eyes, to cleanse wounds	MED: to rinse sore eyes
						Infusion with schnapps, oral application	×	MED: to heal abdominal cramps
						Infusion for inhalation	×	MED: to heal sore throat and bronchitis
						Infusion for bath	COS: to cleanse skin	×
						Decoction, oral application	×	MED: against diarrhea
						Warm chamomile flowers are wrapped in gauze and placed on the chest or eyes	×	MED: to heal bronchitis, to heal cold with respiratory or breathing problems, to heal sore throat and sore eyes
						Maceration in olive oil, topical application	COS: ointment for skin	×
						Ointment made with lard: flowers are fried in lard and drained; topical application	COS: ointment for skin	×
						Fried egg sprinkled with chamomile flowers, oral application	×	MED: treatment of abdominal and menstrual cramps
						Browned flour soup sprinkled with chamomile flowers (as spice)	×	NUT
						Herbal liqueur	NUT/MED	×
					Aerial parts	Infusion, oral application	×	VET: to heal problems of cows after calving and to heal digestive problems
18	7	*Melissa officinalis* L. (Lamiaceae)	Melisa, srčno zelje, štrukljevo perje	C	Leaf	Infusion, oral application	MED: to calm the nerves NUT	MED: to calm the nerves, used before bedtime for better sleep, treatment of stomach problems NUT
						Syrup	NUT	NUT
						Egg omelet with lemon balm leaves, oral application	NUT	MED: treatment of ascites
						Herbal liqueur	NUT/MED	NUT/MED
18	8	*Mentha* spp. (Lamiaceae)	Meta	W/C	Leaf	Infusion, oral application	MED: to calm the nerves, treatment of cold NUT	MED: to calm the nerves, before bedtime for better sleep, treatment of stomach problems, digestive problems, cholagogue NUT
						Syrup	NUT	NUT
						Spice	NUT: spice for meat	×
						Herbal liqueur	NUT/MED	NUT/MED
2	0	*Morus* spp. (*Morus alba* L., *Morus nigra* L.) (Moraceae)	Murva	C	Fruit	Fresh fruits	NUT	×
3	1	*Ocimum basilicum* L. (Lamiaceae)	Bazilika	C	Leaf	Spice	NUT	×
						Infusion	NUT	×
1	0	*Olea europea* L. (Oleaceae)	Oljka	C	Leaf	Infusion	MED: to lower blood pressure	×
8	2	*Origanum majorana* L. (Lamiaceae)	Majaron	C	Aerial parts	Infusion, oral application	NUT	MED: calmative, for better sleeping
						Spice	NUT	NUT
1	4	*Origanum vulgare* L. (Lamiaceae)	Dobra misel	W	Aerial parts	Infusion, oral application	MED: calmative NUT	NUT: children learn better at school
0	2	*Phaseolus vulgaris* L. (Fabaceae)	Fižol	C	Pericarp	Infusion, oral application	×	MED: antidiabetic, antihypertensive
6	10	*Picea abies* (L.) H. Karst. (Pinaceae)	Smreka, smrečje	W/C	Tips	Spruce tip syrup made with sugar or honey, oral application	MED: treatment of cough, sore throat, bronchitis, and cold, to increase the body’s resistance	MED: treatment of cough, cold, bronchitis and sore throat
						Infusion, oral application	Indication was not recorded, although the infusion is used	MED: treatment of cough, good for lungs
					Resin	Pounded resin is wrapped in fabric, topical application	×	MED: treatment of wounds, abscesses and furuncles (the resin pulls the pus out from the inflamed skin), treatment of sore nipples while breastfeeding
3	5	*Pimpinella anisum* L. (Apiaceae)	Janež	C	Fruit	Infusion, oral application	MED: treatment of abdominal cramps, antiflatulent, carminative	MED: treatment of digestive and stomach problems, carminative, antiflatulent VET: treatment of udder inflammation (cow) NUT
						Spice	NUT: spice for beans, sauerkraut, and fried eggs	NUT: spice for pastry
0	2	*Pinus sylvestris* L. (Pinaceae)	Bor	W	Tips	Syrup made from pine tips and sugar, oral application	×	MED: treatment of cough and asthma
					Young cone	Syrup made from pine cones and sugar, oral application	×	MED: treatment of sore throat
10	9	*Plantago* spp. (*Plantago lanceolata* L., *Plantago major* L.) (Plantaginaceae)	Trpotec	W	Leaf	Infusion, oral application	MED: treatment of cough, cold and stomach ulcer	MED: treatment of cough and gastritis NUT
						Syrup, oral application	MED: treatment of cough	MED: treatment of cough
						Fresh leaf is placed on the skin	MED: treatment of wounds	MED: treatment of wounds, furuncles, abscesses and stings
						Herbal liqueur	NUT/MED	NUT/MED
1	6	*Potentilla erecta* (L.) Raeusch (Rosaceae)	Srčna moč	W	Root	Infusion, oral application	MED: treatment of stomach problems	MED: treatment of stomach problems, good for the heart
						Infusion for inhalation	×	MED: treatment of respiratory problems (night asthma)
					Flower and root	Herbal liqueur	NUT/MED	NUT/MED
3	12	*Primula vulgaris* Huds. (Primulaceae)	Trobentica	W	Flower	Infusion, oral application	MED: good for ill children	MED: treatment of cold, good for lungs and respiratory system, diuretic NUT
						Syrup, oral application	×	MED: treatment of cough
					Flower and root	Herbal liqueur	NUT/MED	NUT/MED
4	1	*Prunus avium* L. var. sylvestris (Kirschl.) (Rosaceae)	Divje češnje	W	Fruit	Maceration in schnapps with sugar	NUT	×
						Jam	NUT	×
						Dried fruits	×	NUT: dried fruits for children
0	2	*Prunus domestica* L. (Rosaceae)	Sliva, češplja	C	Fruit	Dried fruits	×	MED: against constipation, good for stool NUT
						Compote from dried fruits	×	MED: against constipation NUT
10	5	*Prunus spinosa* L. (Rosaceae)	Črn trn	W	Flower	Infusion, oral application	×	MED: against blood clotting NUT
					Fruit	Raw fruits	MED: against diarrhea NUT	NUT
						Infusion	NUT	×
						Fruits are macerated schnapps and sugar	NUT	×
0	7	*Pulmonaria officinalis* L. (Boraginaceae)	Pljučnik	W	Aerial parts of flowering plant	Infusion, oral application	×	MED: treatment of pneumonia, cold and cough, good for lungs
						Herbal liqueur	×	NUT/MED
0	1	*Pyrus communis* L. (Rosaceae)	Tepka	C	Fruit	Dried fruit, oral application	×	MED: against diarrhea NUT
2	6	*Quercus* spp. (Fagaceae)	Hrast	W	Bark	Water extract: bark is cooked in water; oral application	VET: against diarrhea (livestock)	×
						Bark is placed in hot water, and a person sits on the steam	MED: treatment of gynecological problems	MED: treatment of bladder inflammation and menstrual problems
					Acorn	Preparation was reported	×	VET: pig feed
5	3	*Robinia pseudoacacia* L. (Fabaceae)	Akacija, ahacovna	W	Flower	Infusion	NUT	NUT
						Fresh flowers cooked with pancake batter in a frying pan	NUT	NUT
						Herbal liqueur	NUT/MED	NUT/MED
22	16	*Rosa* spp. (Rosaceae)	Šipek	W	Fruit	Infusion (dried rose hips can be cooked two or three times)	MED: treatment of cold NUT: contains lots of vitamin C	MED: treatment of cold NUT: contains lots of vitamin C
						Liqueur with schnapps (fruits are macerated schnapps and sugar)	NUT	×
						Jam	NUT	×
11	2	*Rosmarinus officinalis* L. (Lamiaceae)	Rožmarin	C	Leaf, twig	Spice	NUT: spice for goulash	NUT
						Ointment with beeswax, topical application	×	MED: to promote blood circulation (rubefacient)
						Maceration in wine, oral application (a few spoonfuls per day)	MED: against anemia	×
						Cooked in wine, oral application	MED: good for a weak heart	×
						Cooked in wine, topical application	MED: spinal massage for strengthening the spine of children	×
						Infusion, oral application	MED: treatment of sore feet and legs, good for a weak heart	×
						Herbal liqueur	NUT/MED	NUT/MED
2	5	*Rubus idaeus* L. (Rosaceae)	Malina	W/C	Leaf	Infusion	NUT	NUT
						Herbal liqueur	×	NUT/MED
					Fruit	Fresh berries	×	NUT
						Syrup	NUT	×
						Alcoholic distillate from fermented berries	NUT	×
11	8	*Rubus* spp. (only species with black fruits) (Rosaceae)	Robida, kopina	W	Leaf	Infusion, oral application	MED: treatment of diarrhea VET: treatment of diarrhea NUT	MED: treatment of diarrhea NUT
						Fresh leaf is placed on the wound	×	MED: to heal wounds
					Fruit	Fresh berries	NUT	NUT
						Jam	NUT	×
						Herbal liqueur	×	NUT/MED
6	2	*Ruta graveolens* L. (Rutaceae)	Vinska rutica, rüda	C	Aerial parts (leaf)	Maceration in schnapps, oral application	NUT: aperitif	MED: treatment of stomach problems, stimulation of appetite NUT
						Egg omelet with rue	MED: treatment of stomach and digestive problems	×
						Herbal liqueur	NUT/MED	NUT/MED
22	11	*Salvia officinalis* L. (Lamiaceae)	Žajbelj	W/C	Leaf	Infusion for drinking or gargling	MED: treatment of sore throat, cold and cough, good for gums and teeth	MED: treatment of sore throat, inflammation in mouth, cough and cold, good for lungs
						Infusion for inhalation	×	MED: to facilitate breathing
						Leaves cooked in sweet or caramel milk, oral application	MED: treatment of sore throat, cough and bronchitis NUT: before bedtime	MED: treatment of sore throat, cough and cold NUT: before bedtime
						Fresh leaf for chewing	MED: good for teeth and gums, treatment of periodontitis and cold	MED: good for teeth, treatment of inflammation
						Dried leaves, spice	NUT	NUT: spice for black pudding
						Maceration in schnapps and honey, oral application	MED: treatment of cold NUT	×
						Maceration in schnapps, oral application	MED: treatment of cold NUT	NUT
						Maceration in schnapps, topical application	MED: to heal wounds	×
					Flower	Syrup	NUT	×
24	24	*Sambucus nigra* L. (Sambucaceae)	Bezeg	W/C	Flower	Infusion, oral application	MED: treatment of fever, cold, diaphoretic, diuretic, to lower blood sugar level NUT: for thirst in the summer, has good taste	MED: treatment of cold, fever, sore throat, flu, cough and angina NUT: for every day, in the morning, in the evening
						Syrup made by maceration of flowers in water and adding sugar (sometimes called šabesa)	NUT	NUT
						Syrup made with honey; oral administration with teaspoon	×	MED: treatment of cold
						Fresh flowers cooked with pancake batter in a frying pan	NUT	NUT
						Warm compress on the neck	×	MED: treatment of sore throat and cold
						Herbal liqueur	NUT/MED	NUT/MED
					Fruit	Infusion, oral application	NUT: infusion has a strong color, for drinking in the winter	MED: treatment of liver problems, connected with nose bleeding
						Jam	NUT	NUT
						Juice or syrup, oral application	NUT	NUT: contains lots of iron, good for the blood
						Liqueur with Teran wine, made by cooking elderberries in wine	NUT	×
					Leaf	Leaf is placed on painful area (the skin under the leaf must sweat)	MED: treatment of painful or poisoned areas on the skin (for example, on the hand)	×
11	0	*Satureja* spp. (*Satureja montana* L. *Satureja subspicata* subsp. *liburnica* Šilić) (Lamiaceae)	Kraški šetraj, žepek, ožepek, primožek	W	Aerial parts	Spice	NUT: spice for jota (soup), minestrone, vinegar, herb salt, meat, beans	×
6	6	*Sedum maximum* (L.) Suter (Crassulaceae)	Hermelika	W/C	Aerial parts (leaf, flower)	Maceration in schnapps, oral application	MED: treatment of stomach problems NUT: aperitif	MED: treatment of stomach problems NUT: aperitif
						Maceration in schnapps, topical application	MED: treatment of varicose veins	×
					Leaf	Fresh leaf is placed on the skin	MED: to heal wounds	MED: to heal wounds, furuncles, abscesses
						Herbal liqueur	NUT/MED	NUT/MED
7	6	*Sempervivum tectorum* L. (Crassulaceae)	Netresk, natresk	C	Leaf	Juice from the leaf is dripped into ear	MED: treatment of ear inflammation, ear infection and foot and toenail fungus infection	MED: treatment of ear inflammation and ear infection
2	1	*Silybum marianum* (L.) Gaertner (Asteraceae)	Pegasti badelj	C	Seed	Whole or ground seeds, oral application	MED: good for the liver	MED: good for the liver
0	1	*Solanum tuberosum* L. (Solanaceae)	Krompir	C	Tuber	Pieces of potato tuber are wrapped in fabric and lashed on the feet. When the potato becomes black, it pulls the fever out.	×	MED: antipyretic
1	4	*Solidago* spp. (*Solidago virgaurea* L., *Solidago canadensis* L., *Solidago gigantea* Aiton) (Asteraceae)	Zlata rozga	W	Aerial parts of flowering plant	Infusion	MED: good for the prostate	MED: good for bladder, prostate, ovaries
0	3	*Symphytum officinale* L. (Boraginaceae)	Gabez	W	Root	Maceration in schnapps, topical application	×	MED: treatment of rheumatism, to heal wounds
						Maceration in olive oil, topical application	×	MED: to massage inflamed joints (knees)
0	1	*Tamus communis* L. (Discoreaceae)	Blušč	W	Root	Root is placed in animal food, and the animal is rubbed with the root	×	VET: treatment of animals (cows) that have intestinal problems and have become malnourished and cachectic
3	0	*Tanacetum parthenium* (L.) Schultz Bip. (Asteraceae)	Mandrjanca	C	Leaf	Egg omelet	NUT	×
0	2	*Tanacetum vulgare* L. (Asteraceae)	Vratič	W	Flower	Infusion	×	VET: treatment of diarrhea and digestive problems
						Egg and crushed flowers are mixed together and cooked in a frying pan	×	MED: treatment of diarrhea
9	7	*Taraxacum officinal*e agg. F.H. Wigg. (Cichoriaceae)	Regrat, radičkovna, pzdunkula	W	Leaf	Fresh leaves prepared in a salad, sometimes with eggs	NUT: contains lots of iron, good for intestines	NUT: contains lots of iron, counteracts anemia (informants did not eat dandelion leaves for medicinal purposes)
						Dandelion leaves prepared like spinach or prepared together with spinach leaves. Used as soup or side dish to mashed potatoes	×	NUT
					Flower	Infusion	×	NUT
						Syrup, oral application	MED: treatment of sore throat, good for immune system NUT	NUT
					Root	Maceration in schnapps, oral application	×	MED: treatment of stomach problems
						Maceration in schnapps, topical application	×	COS: for lush hair
20	9	*Thymus* serpyllum L. (Lamiaceae)	Materina dušica	W	Aerial parts	Infusion, oral application	MED: good for the heart and respiratory system, treatment of cold and gynecological problems (menstrual cramps) NUT	MED: infusion for breastfeeding mothers NUT
						Spice	NUT	NUT
						Maceration in schnapps	NUT	×
						Herbal liqueur	NUT/MED	NUT/MED
16	20	*Tilia* spp. (*Tilia cordata* Mill. *Tilia platyphyllos* Scop.) (Tiliaceae)	Lipa	C	Flower	Infusion, oral application	MED: treatment of cold, to reduce a fever, diaphoretic NUT	MED: treatment of cold, diaphoretic NUT: for every day, in the morning and evening, has a good taste
						Sleep pillow filled with linden flowers	×	HOUSEHOLD: pillow for sleeping
						Herbal liqueur	NUT/MED	NUT/MED
1	5	*Trifolium* spp. (some informants used species with white flowers and some used species with red flowers) (Fabaceae)	Rdeča detelja, bela detelja, črna detelja	W	Flower	Infusion, oral application	MED: to heal gynecological problems	NUT
4	11	*Tussilago farfara* L. (Asteraceae)	Lapuh	W	Flower	Infusion, oral application	MED: treatment of sore throat and bronchitis, good for lungs	MED: treatment of cold and cough, good for respiratory system
					Leaf	Fresh leaves are wrapped around the legs	×	MED: treatment of leg pain
					Leaf	Cooked leaves	×	VET: pig feed
					Flower and leaf	Herbal liqueur (Jegermajster)	NUT/MED	NUT/MED
13	17	*Urtica dioica* L. (Urticaceae)	Kopriva	W	Leaves on the top, nettle tops	Infusion, oral application	MED: diuretic, to cleanse blood and body, spring and autumn cleansing, counteracts rheumatism, good for the stomach	MED: to cleanse body and blood, diuretic, to cleanse skin, to heal acne, laxative, treatment of diabetes NUT: contains lots of iron
						Infusion for hair rinsing	COS: to cleanse hair	MED: treatment of hair loss COS: for lush hair
						Infusion for bath	×	MED: treatment of tired legs COS: to treat hard skin
						Nettle tops prepared like spinach: soup or side dish to mashed potatoes	NUT	NUT: contains many vitamins and lots of iron, for anemia
					Root	Maceration in schnapps, topical application on scalp	×	COS: to promote good blood circulation of scalp, for lush hair
						Infusion for hair rinsing	COS: for strong hair	×
					Aerial parts	Maceration in water for two days	×	OTHER: fertilizer for the garden
					Aerial parts of young plants	Cooked	VET: feed for pigs	×
2	15	*Vaccinium myrtillus* L. (Ericaceae)	Borovnice	W	Fruit	Infusion from dried blueberries, oral administration	×	MED: treatment of diarrhea VET: treatment of diarrhea (calf)
						Dried fruits for chewing and eating, oral administration	×	MED: treatment of diarrhea
						Compote from dried fruits, oral administration	×	MED: treatment of diarrhea
						Maceration in schnapps, oral administration	MED: treatment of diarrhea NUT	MED: treatment of stomach problems NUT
						Jam	×	NUT
					Leaf	Infusion, oral administration	×	MED: to lower the blood sugar level, treatment of diabetes
						Herbal liqueur	NUT/MED	NUT/MED
1	1	*Valeriana officinalis* L. (Valerianaceae)	Baldrijan	W	Root	Herbal liqueur	NUT/MED	NUT/MED
4	0	*Verbascum densiflorum* Bertol. (Scrophulariaceae)	Lučnik, papeževa sveča	C	Flower	Infusion, oral administration	MED: treatment of sore throat	×
						Maceration in olive oil, topical application	MED: to massage painful joints (rheumatism)	×
						Maceration in schnapps, oral application	MED: treatment of cough and flu, good for lungs	×
1	7	Viola spp. (Violaceae)	Vijolica	W	Flower	Infusion, oral application	NUT	MED: treatment of cough NUT
						Herbal liqueur	NUT/MED	NUT/MED
0	3	*Viscum album* L. (Viscaceae)	Bela omela	W	Leaf	Infusion, oral application	×	MED: antihypertensive, good for the stomach
					Fruit	Glue from cooked fruits applied on a stick	×	OTHER: household agent for catching flies
1	0	*Vitis vinifera* L. (Vitaceae)	Grozdje	C	Fruit	Vinegar for inhalation	MED: inhaled to prevent cold	×
0	1	*Zea mays* L. (Poaceae)	Koruza	C	Silk	Infusion, oral application	×	MED: treatment of urinary tract inflammation

On average, the informants reported 20 different plants per interview in both regions (Karst: standard deviation 5.9, max. 30, min. 9; Gorjanci: standard deviation 10.4, max. 42, min. 6). Altogether, the informants made 493 reports of collected plants in Karst and 490 in Gorjanci. The informants in both regions reported 47 wild plants, 12 plants that grow wild and can be cultivated and 35 cultivated plants, although they were asked to list the plants that they collected in the wild. Listing of cultivated plants instead of plants collected in the wild has also been observed in other studies [38, 54]; it is likely that informants mentally link the reported wild-collected plants to homemade remedies and then remember other plants that are also used for home remedies, although they are cultivated. It is also possible that they cultivate some plants that are primarily found in the wild [38].

The important plants in each region (Table 4)

**Table 4:** List of plants reported by at least 3 informants in Karst or Gorjanci, with their frequency, rank and Smith’s salience index

Karst				Gorjanci			
Plant	Freq.	Rank	Smith’s S	Plant	Freq.	Rank	Smith’s S
*Sambucus nigra*	24	4.5	0.794	*Sambucus nigra*	24	6.38	0.694
*Salvia officinalis*	22	11.45	0.448	*Achillea millefolium*	20	10	0.462
*Rosa* spp.	22	10.36	0.491	*Tilia* spp.	20	7.65	0.541
*Thymus serpyllum*	20	7.05	0.552	*Matricaria chamomilla*	19	10.05	0.465
*Mentha* spp.	18	8.22	0.458	*Urtica dioica*	17	8.24	0.398
*Melissa officinalis*	18	8.17	0.475	*Hypericum perforatum*	16	7.44	0.478
*Matricaria chamomilla*	16	11.44	0.329	*Rosa* spp.	16	17.25	0.200
*Tilia* spp.	16	7.13	0.439	*Centaurium* spp.	15	11.07	0.376
*Achillea millefolium*	14	10.29	0.328	*Vaccinium myrtillus*	15	14.67	0.259
*Urtica dioica*	13	9.85	0.300	*Fragaria vesca*	13	11.92	0.257
*Crataegus* spp.	13	9.08	0.335	*Arnica montana*	12	14.08	0.227
***Foeniculum vulgare***	13	12.69	0.241	*Calendula officinalis*	12	11.25	0.296
*Juniperus communis*	12	12.5	0.225	*Primula vulgaris*	12	8.5	0.301
***Satureja*** spp.	11	9.64	0.252	*Tussilago farfara*	11	11.09	0.235
*Centaurium* spp.	11	12.36	0.216	*Salvia officinalis*	11	10.45	0.245
*Hypericum perforatum*	11	11.45	0.243	*Equisetum arvense*	10	14.8	0.203
*Rosmarinus officinalis*	11	11.36	0.224	*Betula pendula*	10	16.3	0.158
*Rubus* spp.	11	12.64	0.202	*Picea abies*	10	13.2	0.200
*Prunus spinosa*	10	11.7	0.207	*Thymus serpyllum*	9	9.22	0.228
*Plantago* spp.	10	11.4	0.208	*Juglans regia*	9	20.67	0.096
*Laurus nobilis*	9	16.56	0.087	*Juniperus communis*	9	19.33	0.121
*Taraxacum officinale agg*.	9	10	0.178	*Plantago* spp.	9	12.89	0.192
*Calendula officinalis*	9	10.78	0.197	*Rubus* spp.	8	15.38	0.157
*Cornus mas*	8	13.5	0.149	*Mentha* spp.	8	8	0.229
*Origanum majorana*	8	9.13	0.182	*Viola* spp.	7	9.86	0.178
*Artemisia* spp.	7	14.14	0.122	*Taraxacum officinale* agg.	7	21.86	0.095
*Lavandula* spp.	7	11.14	0.162	*Carum carvi*	7	12.71	0.166
*Sempervivum tectorum*	7	16	0.096	*Melissa officinalis*	7	8.43	0.209
*Picea abies*	6	13.33	0.080	***Pulmonaria officinalis***	7	11.29	0.184
***Asparagus*** spp.	6	12.5	0.079	*Sempervivum tectorum*	6	13.83	0.110
*Althaea officinalis*	6	13.67	0.091	*Potentilla erecta*	6	18.83	0.097
*Ruta graveolens*	6	18.67	0.077	*Sedum maximum*	6	18	0.088
*Sedum maximum*	6	12.67	0.103	*Crataegus* spp.	6	7.83	0.167
*Arnica montana*	6	12.17	0.108	*Artemisia* spp.	6	22.67	0.068
*Robinia pseudacacia*	5	20.4	0.028	*Quercus* spp.	6	19	0.077
*Fragaria vesca*	5	15.4	0.087	*Trifolium* spp.	5	11.6	0.106
***Aloysia citrodora***	5	13	0.073	*Pimpinella anisum*	5	9.4	0.138
***Prunus avium***	4	17.25	0.016	*Prunus spinosa*	5	12.2	0.109
*Tussilago farfara*	4	15.5	0.069	*Rubus idaeus*	5	12.8	0.100
*Anthyllis vulneraria*	4	8.25	0.117	*Bellis perennis*	4	12	0.104
*Levisticum officinale*	4	13	0.07	*Solidago* spp.	4	13	0.085
***Verbascum densiflorum***	4	15.25	0.081	*Origanum vulgare*	4	9.75	0.068
*Ocimum basilicum*	3	10.33	0.061	*Castanea sativa*	4	17.25	0.055
*Gentiana lutea*	3	14	0.055	*Allium ursinum*	4	17.25	0.035
*Aesculus hippocastanum*	3	8.67	0.067	***Symphytum officinale***	3	13.33	0.055
*Carum carvi*	3	18.67	0.022	***Berberis vulgaris***	3	9.33	0.060
***Tanacetum parthenium***	3	17.33	0.010	***Viscum album***	3	24	0.028
*Pimpinella anisum*	3	12.33	0.061	***Linum usitatissimum***	3	23	0.029
*Primula vulgaris*	3	16	0.054	*Robinia pseudacacia*	3	25	0.038
				***Galium odoratum***	3	26	0.028

Plant species that were reported in only one of the two regions are printed in bold

were mentioned frequently and were mentioned early in the interview (low rank); the frequency and rank were significantly correlated in both Gorjanci and Karst (Pearson’s correlation *p* = 0.0007 and *p* < 0.00001, respectively). The important plants had also a high Smith’s salience index, which quantifies the importance of a plant in relation to its frequency and order of mention (rank) in free-listing [52]. Many plants were distinctive for one or a few informants. In each region, approximately 64% of the plants were mentioned by fewer than 7 informants, and approximately 36% of the plants were mentioned by at least 7 informants. The informants mentioned 49 plants in Karst and 52 in Gorjanci that had a frequency of 1 to 6; in addition, they mentioned 28 plants in Karst and 29 in Gorjanci that had a frequency of 7 to 24 (Table 3). The above-mentioned results show that some plants were mentioned very frequently, and many plants were mentioned by a few informants. The frequency of mention decreased gradually, and there was no noticeable break in the frequency of mention between plants mentioned by many informants and plants mentioned by just a few informants; therefore, a small group of important plants for each region could not be obtained [48].

The most frequently reported plant was *Sambucus nigra*; 24 informants in Karst and 24 informants in Gorjanci (K: 24; G: 24) reported use of this plant. *S. nigra* has also been frequently reported in northern Italy [55] and in some parts of Austria [37, 56], Croatia [57, 58] and Bosnia and Herzegovina [59]. Other frequently reported plants were *Rosa* spp., *Salvia officinalis*, *Thymus serpyllum*, *Mentha* spp., *Melissa officinalis*, *Matricaria chamomilla*, and *Tilia* spp. in Karst and *Achillea millefolium*, *Tilia* spp., *Matricaria chamomilla*, *Urtica dioica*, *Hypericum perforatum*, *Rosa* spp., *Centaurium* spp., and *Vaccinium myrtillus* in Gorjanci. The above-mentioned plants were reported by at least 15 informants in one region (Table 4). These plants belong to the European ethnomedicinal flora; some of them (e.g., *Sambucus nigra* and *Urtica dioica*) are common and abundant wild species with a wide distribution area and frequent use, while others are widely used cultivated plants, e.g., *Matricaria recutita* and *Tilia* spp. [60].

In a comparison of plants reported in Slovenia and those reported in ethnobotanical studies in Austria, Serbia and Bosnia and Herzegovina, many similarities were observed. Many plants reported in our study were also reported in Austria: 22 out of 27 wild-collected food plants in a hilly area in Styria [61], 48 out of 76 wild or cultivated plants reported in the Alpine valley Grosses Walsertal [37], and 28 out of 64 wild-collected plants in Kartitsch (eastern Tyrol) [54]). Approximately half of the listed plants in Serbia were also reported in Slovenia: 46 out of 69 in southwestern Serbia [62], 23 out of 45 in Mt. Rtanj (eastern Serbia) [63], 60 out of 128 on Suva Planina mountain (southeastern Serbia) [64], and 38 out of 83 on Kopaonik Mountain (central Serbia) [65]. Similar use of plants was also observed in Bosnia and Herzegovina: 76 out of 254 wild and cultivated medicinal plants in eastern, northern and northeastern Bosnia and Herzegovina [59]; and 73 out of 228 wild and cultivated medicinal plants in central, southern and western Bosnia and Herzegovina [66]. We did not observe any important plants used in any of the two studied areas that had not been previously reported in the above-mentioned neighboring areas.

While the focus of our study was the medicinal use of plants, the informants did not make a clear distinction between medicinal and nutritive uses and reported a broad spectrum of uses. We classified these uses into several categories. The most frequently reported uses of plants were medicinal and nutritive: 81 plants were used as medicine, and 63 plants were used as food. Purely medicinal use was reported for 29 plants, purely nutritive use was reported for 11 plants, and a combination of medicinal and nutritive uses was reported for 52 plants. Other uses were less frequently reported: 17 plants were used for animal healthcare and 8 for cosmetic use. These uses were almost always mentioned together with nutritive or medicinal use.

### Comparison of plants reported in Karst and Gorjanci

**Fig. 2:**
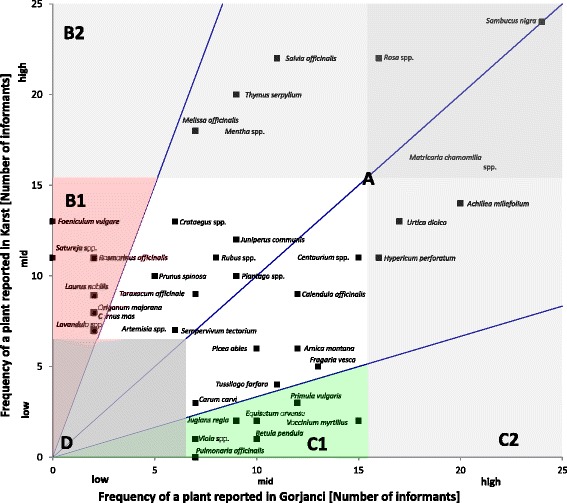
Comparison of the frequency distribution of plants reported by 25 informants in Gorjanci and 25 informants in Karst. Frequencies below 7 are considered low, those from 7 to 15 mid, and those from 16 to 25 high. Plants that were reported by at least 7 informants in one region are represented by a *dot* with the plant’s scientific name. Due to the high number of plants, those with frequencies lower than 7 in both regions are not represented by a *dot*; they lie in section *D*. The *middle blue line* represents plants with equal frequencies in both regions. The other *blue lines* represent plants with frequencies that were threefold higher in one region than in the other

A comparison of the frequency distributions of the plants reported in Karst and Gorjanci (Fig. 2) showed that there were plants reported with similar or different frequencies in both regions. The 54 plants with a low frequency (mentioned by fewer than 7 informants in both regions) (Fig. 2, section D) were not included in this comparison. The plants whose frequency in one region was 3-fold higher than that in the other region were considered typical for that region. The plants that were typical for Karst were *Foeniculum vulgare*, *Satureja* spp., *Rosmarinus officinalis*, *Laurus nobilis*, *Cornus mas*, *Origanum majorana* and *Lavandula* spp. (Fig. 2, section B1). The plants that were typical for Gorjanci were *Vaccinium myrtillus*, *Primula vulgaris*, *Equisetum arvense*, *Betula pendula*, *Juglans regia*, *Pulmonaria officinalis* and *Viola* spp. (Fig. 2, section C1). Of the plants with a high frequency (mentioned by more than 15 informants in at least one region), no plants were typical for only one region (Fig. 2, sections B2 and C2). In both regions, 25 plants were reported equally or up to 3-fold higher in one region than in the other (Fig. 2, plants in section A). These plants were considered regionally non-typical.

*Laurus nobilis*, *Rosmarinus officinalis*, and *Origanum majorana* were typical for Karst but were also reported in Gorjanci. These plants are part of the Mediterranean flora and are somewhat cold sensitive; therefore, they are more widely and easily cultivated in house gardens in Karst due to suitable growing conditions. They were mostly used as kitchen spices. *Satureja* spp., *Cornus mas* and *Foeniculum vulgare* were also typical plants for Karst and were not reported in Gorjanci. They grow wild in Karst but not in Gorjanci [67]. *Foeniculum vulgare* was also sometimes cultivated in house gardens in Karst for easier accessibility. *Lavandula* spp. was also typical for Karst. It was mostly used as an ornamental plant, but two informants in Karst reported that they were cultivating *Lavandula* spp. in fields and wanted to start producing lavender essential oil. It seems that *Lavandula* spp. has become an interesting plant for small businesses in Karst in recent years due to suitable growing conditions [68]. Therefore, it is very likely that *Lavandula* spp. was more frequently reported in Karst than in Gorjanci due to this plant’s growing popularity. Guštin Grilanc also reported the use of the above-mentioned seven plants in Karst [30], but Lokar and Poldini reported only the use *Satureja montana* in Trieste Karst [29].

*Vaccinium myrtillus*, *Equisetum arvense*, *Betula pendula* and *Pulmonaria officinalis* were typical for Gorjanci and were rarely or not mentioned in Karst. This finding is probably because they grow wild in Gorjanci but are scarce in Karst [67]. Makarovič reported the use of the above-mentioned plants in Dolenjska and Bela Krajina [32]. Lokar and Podini reported only the use of *Pulmonaria officinalis* in Trieste Karst [29], and Guštin Grilanc reported only *Equisetum arvense* in Karst [30].

*Primula vulgaris* and *Viola* spp. have a wide distribution area in Slovenia and grow in both research areas (Karst and Gorjanci) [67]. Therefore, it was unexpected that these plants were observed to be typical for Gorjanci in our research. Makarovič did not report their use in Dolenjska or Bela Krajina [32], but Guštin Grilanc reported the use of both plants in Karst; Lokar and Poldini also reported the use of *Primula vulgaris* in Trieste Karst [29].

This comparison indicates that there is a relatively homogeneous tradition of medicinal plant use in both areas, with minor regional differences. The regional differences for wild-collected plants are mostly due to ecological availability (all plants were not ubiquitously available in Karst and Gorjanci). The importance of ecological availability for comparison was previously shown in a comparative study of wild food plant consumption in Italy [55]. In addition to ecological availability, suitable growing conditions (climate and soil) were also important or essential for the cultivation of medicinal plants. In this regard, vicinity to the Adriatic Sea and Mediterranean flora plays a major role for Karst; in contrast, the continental climate in Gorjanci prevents the cultivation of cold-sensitive plants. We did not find that cultural differences played a role in the regional differences in the use of plant species.

### Medicinal use of plants in Karst and Gorjanci

#### General data on the medicinal use of plants

The plants that were reported in Karst and Gorjanci were grouped into 8 categories according to their therapeutic use (Table 5)

**Table 5:** Categories of medicinal use of plants in Karst and Gorjanci with the number of reports and plants in each region

Disease or action of the medicine	No. of plants reported in Karst	No. of reports in Karst	No. of plants reported in Gorjanci	No. of reports in Gorjanci	No. of plants reported in Karst and Gorjanci	No. of reports in Karst and Gorjanci
**Respiratory system disorders**	17	58	19	88	26	146
Cold	9	28	12	33	16	61
Cough	5	9	12	21	14	30
Sore throat	7	12	6	14	10	26
“Good for lungs” and “good for respiratory system”	3	4	5	8	7	12
Bronchitis	4	4	2	3	5	7
Flu	1	1	3	3	4	4
Asthma	0	0	2	2	2	2
Breathing problems	0	0	2	2	2	2
Pneumonia	0	0	1	1	1	1
Angina	0	0	1	1	1	1
**Gastrointestinal disorders**	18	50	25	87	29	137
Carminative (digestive problems, spasms, stomach pain, and flatulence)	16	43	21	67	25	110
Against diarrhea	4	6	7	15	9	21
Appetite stimulation	1	1	2	2	3	3
Laxative	0	0	2	3	2	3
**Skin diseases**	12	35	14	59	20	94
Wounds	10	17	8	30	14	47
Burns and sunburns	3	7	3	10	3	17
Furuncles and abscess	0	0	4	7	4	7
Warts	2	3	1	2	2	5
Contusions and bruises	3	3	2	2	3	5
Rash and eczema	1	1	2	2	2	3
Rubefacient	1	2	1	1	2	3
Scabs	1	1	1	1	2	2
Stings	0	0	2	2	2	2
Snake bites	1	1	0	0	1	1
Sweaty feet	0	0	1	1	1	1
Inflamed nipples	0	0	1	1	1	1
**Genitourinary system disorders**	9	24	11	39	14	63
Diuretic, “cleansing of body, kidneys and blood”	4	14	6	22	8	36
Gynecological problems	5	10	5	8	6	18
Urinary tract inflammation	0	0	5	9	5	9
**Miscellaneous infections**	6	24	12	32	13	56
Ear infection	1	7	1	5	1	12
Fever	2	2	7	8	7	10
Mouth inflammation	1	5	2	5	2	10
Eye infection	2	1	2	8	3	9
Diaphoretic	2	5	1	3	2	8
Anthelmintic	1	3	2	3	2	6
Fungal nail infection	1	1	0	0	1	1
**Musculoskeletal system disorders**	9	15	11	20	14	35
Muscle or joint pain in various body parts	4	5	2	6	5	11
Rheumatism	4	5	4	7	6	11
Pain in legs	2	2	4	4	6	6
Pain in the back	2	2	0	0	2	2
Pain in hands	0	0	1	1	1	1
Weak spine of children	1	1	0	0	1	1
Knee effusion	0	0	1	1	1	1
Swollen legs	0	0	1	1	1	1
**Nervous system disorders**	6	18	5	17	9	35
Calmative	5	**15**	5	11	7	26
Sleep disorders	1	2	3	5	4	7
Epilepsy	1	1	0	0	1	1
Depression	0	0	1	1	1	1
**Heart, blood and circulatory system disorders**	8	21	6	9	13	30
Heart trouble	3	13	2	4	4	17
Varicose veins	3	4	1	1	3	5
High blood pressure	1	1	2	3	3	4
Anemia	2	2	0	0	2	2
Hemorrhoids	1	1	0	0	1	1
Against blood clotting	0	0	1	1	1	1

Groups of diseases are labeled in bold

. The highest number of plants was used for the treatment of gastrointestinal disorders, respiratory system disorders and skin diseases. These categories also had the highest number of use reports. This finding is comparable to recent reports from southern Italy [40–42] and to reports from Adam Fisher, who collected traditional uses of plants in western Ukraine in the 1930s [69]. However, the distribution of uses in Bosnia and Herzegovina was slightly different, with urogenital indications being the most frequent [66, 70]. The differences may be due to different methodologies or conditions in the region. The informants also reported using plants for genitourinary system disorders, miscellaneous infections, and musculoskeletal system disorders. The informants made the lowest number of reports for the treatment of heart, blood and circulatory system disorders, and they used the lowest number of plants to treat nervous system disorders. For different diseases, we observed different ratios between the number of reports and the number of plants: e.g., for the treatment of ear infection, we obtained 12 reports on the use of only one plant (in both regions); in contrast, 10 informants reported 7 different plants for the treatment of fever and 6 informants reported 6 different plants for the treatment of leg pain.

#### Differences in the medicinal use of plants in Karst and Gorjanci

Differences in the reported medicinal use (indication) of some frequently collected plants were observed between Karst and Gorjanci. These regional differences could be explained by cultural differences, which were possibly due to proximity to neighboring areas. *Matricaria chamomilla* was used by 16 informants in Karst and 19 in Gorjanci. While most informants in Karst reported its use as a sedative (calmative) (K: 7; G: 1), most of the Gorjanci informants reported its use for the treatment of respiratory infections (cold, flu, bronchitis, sore throat, or cough) (K: 1; G: 6), sore eyes (K: 2; G: 9), dysmenorrhea (K: 1; G: 4) or as a carminative (K: 5; G: 13). The difference between the regions was statistically significant (Fisher’s exact test *p* = 0.010). Lokar and Poldini also reported that *M. chamomilla* was used as a sedative in Trieste Karst and Istria [29], but Guštin Grilanc did not report this use in Karst [30]. Similarly, some ethnobotanical studies in Austria and Croatia reported that *M. chamomilla* was used as a sedative [56, 71], but some did not [37, 72]. *M. chamomilla* was widely reported as a sedative in Italy [40, 73, 74].

Altogether, 25 different plants were used as a carminative: 16 in Karst and 21 in Gorjanci. The treatment of poor digestion, spasms, stomach pain, and flatulence (carminative) was more frequently reported with *Juniperus communis* (G: 5; K: 9) in Karst and with *Juglans regia* (G: 6; K: 0), *Centaurium* spp. (G: 12; K: 5) and *M. chamomilla* (G: 13; K: 5) in Gorjanci; this difference was statistically significant (Fisher’s exact test *p* = 0.024). Lokar and Poldini did not mention the medicinal use of *J. communis* or *J. regia* in Trieste Karst, but they reported the medicinal use of infusion of *Centaurium erythraea* as a bitter tonic in Istria and as a decoction for the treatment of gastritis in Trieste Karst. In that study, decoction of *Satureja montana* was reported as a bitter tonic in Trieste Karst and alcoholic macerate as a tonic aromatic in Trieste Karst and Istria [29]; in our study, the informants in Karst reported only the nutritive use of *Satureja* spp.

Among the 8 medicinal plants that were reported for the treatment of diarrhea, *Vaccinium myrtillus* was the only plant that was reported by more than two informants. Its antidiarrheal use was reported by 10 informants, and most of the reports were from Gorjanci (K: 1; G: 9). *V. myrtillus* was more frequently mentioned in Gorjanci than in Karst (G: 15; K: 2), which is probably due to the scarcity of *V. myrtillus* in Karst and its presence in Gorjanci [67]. In the previous studies, the use of *V. myrtillus* was also not reported in Karst [30] or Trieste Karst [29] but was reported in Dolenjska and Bela Krajina, where it was also used for the treatment of diarrhea and stomachache [32].

*Sambucus nigra* was the most frequently mentioned plant (K: 24; G: 24). While most of the informants in Gorjanci reported its use for the treatment of respiratory infections (cold, flu, angina, or high fever, and as a diaphoretic) (G: 14; K: 5), most of the informants in Karst used it merely for nutritive purposes (G: 10; K: 18); this difference was statistically significant (Fisher’s exact test *p* = 0.017). Lokar and Poldini also did not report any medicinal use of *S. nigra* in Trieste Karst [29], but Guštin Grilanc reported its use against cough and cold in Karst [30]. In Dolenjska and Bela Krajina, its use was reported for sore throat, cough, cold, and high fever [32].

*Salvia officinalis* was reported in both regions for gargling and for the treatment of sore throat, mouth infection, and mouth inflammation (K: 11; G: 9) but was more frequently reported for the treatment of colds in Karst than in Gorjanci (K: 9; G: 2). *S. officinalis*, *S. nigra*, and *Tilia* spp. were the most frequently reported plants used for the treatment of colds. *Tilia* spp. was reported in both regions (K: 7; G: 4); *S. officinalis* was more frequently reported in Karst (K: 9; G: 2); and *S. nigra* was more frequently reported in Gorjanci (G: 11; K: 3). *Rosa* spp. was seldom reported for the treatment of colds (G: 3; K: 2). Its use was more often reported merely due its nutritive value (G: 19; K: 12), and some informants reported that it was beneficial due to its (high) content of vitamin C (G: 7; K: 6).

#### Continuity between nutritive and medicinal use

In a number of plants, medicinal and nutritive uses were interconnected because approximately 50% of the plants were reported for both medicinal and nutritive purposes. Frequently, the same informant reported medicinal and nutritive uses of the same plant, plant part and plant preparation. In addition, properties that promote health, e.g., a high content of vitamin C, were also reported for plants. There seems to be a continuous passage between the nutritive and medicinal uses of plants since it is the informant’s intended use that determines whether the plant is a food or a medicine.

Four plants were reported for use in association with the urinary tract: *Urtica dioica*, *Asparagus* spp., *Equisetum arvense* and *Betula pendula*. A comparison of their use shows that some plants were more often reported as food, and some were more commonly used for the treatment of urinary infections (Table 6)

**Table 6:** Comparison of the reported uses of plants that were collected in Karst and Gorjanci

Plant	No. of informants		Reported uses in Karst and Gorjanci		
	Karst	Gorjanci	Food	Cleansing of the body, blood and kidneys; diuretic properties	Treatment of urinary infections
*Urtica dioica*	13	17	14	15	0
*Asparagus* spp.	6	0	6	5	0
*Equisetum arvense*	2	10	2	10	10
*Betula pendula*	0	6	0	6	6

. For the above-mentioned plants, the informants also reported properties such as cleansing of the body, blood and kidneys and diuretic properties, which were mentioned together with purely nutritive or purely medicinal uses. Some plants were predominantly used for one purpose or the other. Two informants who used *E. arvense* for nutritive purposes added it to an herbal liqueur made from many plants (“Jegermajster”).

The phenomenon of people using the same plants as food and as medicine has been observed in many cultures [75–78]. In theory, the basic characteristics of plants that influence their categorization are their nutritive value and medicinal or health benefits, which are beyond basic nutrition [79]. In practice, some constituents (e.g., vitamin C) have both nutritive and therapeutic benefits, and some plants that do not contain important nutrients are used as food (e.g., as herbal tea for refreshment). Furthermore, whether a plant is consumed as food or as medicine is also based on local beliefs, perceptions and intention of use [78]. The case of garlic, which became a legal matter in the European Court, demonstrates that the border between the use of a plant as a food and medicine can be unclear [80].

### Medicinal plants in animal healthcare

Studies of ethnoveterinary medicine have been conducted in many European countries, and they are important for the preservation of traditional knowledge and the identification of alternatives for the treatment of animal diseases [81–84]. Our research did not focus on ethnoveterinary medicine, but a small number of medicinal plants used for animal health and welfare were reported in the interviews. The informants in Gorjanci and Karst mentioned the use of 14 plants in animal healthcare and three as animal feed (Table 3). The most frequently reported medicinal plants for animals were *Matricaria chamomilla* (K: 0; G: 6) and *Quercus* spp. (K: 1; G: 4); other plants were reported by one or two informants. In Gorjanci, infusion of *M. chamomilla* was reported for the treatment of digestive problems in livestock; it was also given to cows after calving. The bark of *Quercus* spp. was reported for the treatment of diarrhea in livestock. *M. chamomilla* and Quercus spp. are among the most frequently mentioned species in European ethnoveterinary studies [83].

Most of the plants were reported for the treatment of gastrointestinal problems: in addition to the bark of *Quercus* spp., dried fruits of *Vaccinium myrtillus* (in calves), flowers of *Tanacetum vulgare* and leaves of *Rubus* spp. (species with black fruits) (in livestock) were reported for diarrhea; the infusion of flowers and herbs of *Centaurium* spp. (in livestock), roots of *Gentiana lutea* (in livestock), flowers of *T. vulgare*, flowers and herbs of *M. chamomilla* (in livestock) and schnapps macerate of *Juglans regia* were reported for the treatment of digestive problems; the oil macerate of *Hypericum perforatum* flowers was reported for the treatment of indigestion in hens that had eaten too much sand; *Artemisia* spp. was reported for the treatment of low appetite in livestock; and the root of *Tamus communis* was reported for the treatment of a cow with intestinal problems that became malnourished and cachectic. The infusion of *Pimpinella anisum* fruits was reported for the treatment of mastitis (udder inflammation) in cows. An informant in Gorjanci reported the use of bread with a few drops of essential oil from fermented berry cones of *Juniper communis* to treat a sick bullock, which immediately felt better and could stand up. Water extract from cooked leaves of *J. regia* was applied to a bullock’s skin to protect the animal from horseflies. A schnapps macerate and a water infusion of *Inula hirta* flowers were reported for the treatment of a dog with a snake bite. Aerial parts of *Cannabis sativa*, acorns of *Quercus* spp. and cooked leaves of *Tussilago farfara* were reported as pig feed.

The use of nine medicinal plants was the same in humans and animals. In the case of *Inula hirta* and *Juniperus communis*, the informants illustrated and augmented their claims for the effectiveness of the medicinal plant with an example of treating a sick animal with the preparation of that plant. Similarly, an informant from Gorjanci emphasized the high medicinal value of *Centaurium* spp. by reporting that the animal (cow) was healthier if it fed on grass containing centaury. This statement could also be understood to mean that feeding is vital for animal welfare [84]. We did not observe the use of any important plants in animals, which was not previously reported in the above-mentioned literature.

### Medicinal preparations

Informants in Karst and Gorjanci reported a high number of medicinal preparations for oral and topical applications (Tables 7 and 8)

**Table 7:** Medicinal preparations for oral application reported in Karst and Gorjanci

Preparation method		No. of plants
Fresh and dried plant material	Dried fruits	3
	Raw fruits	1
	Juice	1
	Leaf for chewing	1
	Seeds	1
	Bulb	1
Heat-processed plant material	Infusion	48
	Decoction	1
	Compote from dried fruits	2
	Leaves cooked in (caramel) milk	2
	Cooked in wine	1
	Roux soup with eggs and flowers	1
	Egg omelet with plant material	3
	Eggs cooked in a frying pan and sprinkled with flowers	1
Distillation	Essential oil	1
	Alcoholic distillate	1
Maceration	Maceration in schnapps	16
	Maceration in wine	2
	Syrup	9

**Table 8:** Medicinal preparations for topical application reported in Karst and Gorjanci

	Preparation method		No. of plants
Skin	Fresh plant material	Fresh plant material is placed on the skin	10
		Latex	2
		Resin	1
	Heat-processed plant material	Ointment made with lard	2
		Fried plant material	1
		Poultice made from cooked plant material	2
		Infusion for warm compress	1
		Infusion	2
		Infusion for bath	3
		Cooked in wine	1
	Distillation	Essential oil	1
		Alcoholic distillate	1
	Maceration	Maceration in oil	5
		Maceration in schnapps	10
Eyes	Heat-processed plant material	Infusion for eye rinsing and compress	1
	Fresh plant material	Seed is placed in eye	1
Lungs	Heat-processed plant material	Infusion for inhalation	3
	Fermentation	Vinegar for inhalation	1
Ears	Fresh plant material	Juice is dripped into ear	1

. However, the large number of preparations does not reflect the relevance of use, as some preparations were reported by all informants and some by a single informant. The list merely reflects the versatility of the preparations described in the interviews.

For oral application, informants reported 15 different methods of preparing the plant material. Most of the methods included heat processing or maceration; the use of unprocessed fresh or dried plants was reported for only eight plants. Infusions, alcoholic macerates and syrups were the prevailing preparations for many of the reported plants; the remaining 12 preparations were specific for one to three plants (Table 7). Most of the preparations for topical application were reported for skin, and only a few preparations were reported for eyes, ears or lungs (Table 8). The large number of different skin preparations might be associated with the considerable number of plants reported for skin diseases, as 20 were reported in both regions (Table 5). The plants that were used for skin were mainly prepared by maceration in oil or schnapps, or fresh plant material was applied to the skin.

Alcoholic distillate (“brinjevec”) and essential oil (“brinjevo olje”) of *Juniperus communis* are of special interest because they have likely been known for centuries in the territory of Slovenia. “Brinjevec” is a protected spirit drink with a geographical indication; it was mentioned in 1689 by Janez Vajkard Valvasor in *Die Ehre dess Hertzogthums Crain* and is made by distilling fermented juniper berries from *J. communis*. The side product of this distillation is an essential oil [85]. The medicinal use of the essential oil was reported in both regions; it was used internally and externally. The external use of distilled oil from *J. communis* was reported by Istro-Romanians in Žejane, northeastern Istria, Croatia [71]; Croatians living Čičarija, northern Istria, reported the internal use of the alcoholic distillate [72].

In Karst and Gorjanci, five women aged 65 to 87 years reported the medicinal use of three common Slovenian dishes: browned flour soup (“prežganka” in Slovene), egg omelet (“omleta” in Slovene; “frtalja” in Karst), and eggs cooked in a frying pan. The dishes were used medicinally for the treatment of gastrointestinal problems and dysmenorrhea when prepared with the following plants: *Achillea millefolium*, *Ruta graveolens*, *Tanacetum vulgare* and *Matricaria chamomilla* (Table 3). This information might be of special interest since the concomitant use of these plants and food (eggs) might alter the body’s response to the medicine compared to using an infusion of the plant.

Two women aged 79 and 87 years from villages in the foothills of Gorjanci reported several unusual medicinal preparations made from plant, fungal, and animal materials: a necklace made from garlic cloves (*Allium sativum*) was used as an anthelmintic; pig feces was used to heal furuncles and abscesses; humane urine was used to disinfect wounds; pork cracklings (fried bacon cubes in lard) were used to heal scabs; snails were used to heal warts; and sour yeast (known as “kravajc” in Gorjanci) made from corn flour, millet chaff and wine foam was used to treat pain. A small loaf of “kravajc” was wetted with warm water and lashed on the sole of the foot to relieve foot pain (“kravajc pulled the pain out of the foot”).

A few informants (K: 1; G: 3) reported the preparation of herbal liqueurs. They collected different plants from spring to autumn and macerated them in homemade schnapps. The informants in Gorjanci called this liqueur “jegermajster” (similar to the name of the commercial aperitif Jägermeister), and the informants in Karst called it “bitter”. It was used for nutritive purposes and to treat digestive problems and stomachaches. The informants reported using the following plants for the liqueur: *Achillea millefolium*, *Aesculus hippocastanum*, *Althaea officinalis*, *Anthyllis vulneraria*, *Arnica montana*, *Artemisia* spp., *Bellis perennis*, *Calendula officinalis*, *Castanea sativa*, *Centaurium* spp., *Equisetum arvense*, *Fragaria vesca*, *Galium odoratum*, *Hypericum perforatum*, *Juglans regia*, *Juniperus communis*, *Matricaria chamomilla*, *Melissa officinalis*, *Mentha* spp., *Plantago* spp., *Potentilla erecta*, *Primula vulgaris*, *Pulmonaria officinalis*, *Robinia pseudacacia*, *Rosmarinus officinalis*, *Rubus idaeus*, *Rubus* spp. (species with black fruits), *Ruta graveolens*, *Sambucus nigra*, *Thymus serpyllum*, *Tilia* spp., *Tussilago farfara*, *Vaccinium myrtillus*, *Valeriana officinalis*, and *Viola* spp. (Table 3).

### Sources of knowledge about plants

The transmission of knowledge about medicinal plants (which includes behaviors, attitudes, or technologies) is a complex process. We can distinguish between different transmission processes between individuals, e.g., from parent to child and between members of the same generation [86, 87]. Ethnobotanical studies have mainly focused on individuals as transmitters of knowledge; however, in literate societies, media such as books, television, journals and the internet are also important [88, 89] since this type of transmission can bring very rapid cultural change [87].

In Slovenia, many books were published about medicinal plants in the twentieth century, and they could easily influence the folk knowledge about plants. A general question, “Where did you get your knowledge about plants?” was posed to the informants to determine whether books or other media had influenced their knowledge. Media as non-oral sources were important for the botanical knowledge of the informants, as 20 informants in Karst and 12 in Gorjanci reported them. Books were frequently reported in both regions, whereas television, magazines, newspapers, the internet and radio were rarely reported (Table 9)

**Table 9:** Sources of knowledge about plants for informants in Karst and Gorjanci

		No. of informants in Karst	No. of informants in Gorjanci
Oral sources - relatives	Mother	8	9
	Father	0	2
	Parents	0	2
	Grandmother	5	5
	Grandfather	0	1
	Grandparents	1	0
	Sister	1	0
	Brother	0	1
	Aunt	1	0
	Uncle	1	0
	Mother-in-law	1	3
	Sister-in-law	0	1
	Cousin (female)	0	1
	Relatives (in general)	1	0
Oral sources - non-relatives	Neighbors	0	3
	Neighbor - woman	0	2
	Neighbor - man	1	0
	Friends	1	0
	Woman friend	3	0
	Co-worker - woman	2	2
	Older women	0	2
	Older people	0	1
	Doctor - woman	0	1
	Doctor - man	1	0
	Veterinarian - man	0	1
	Herbalist - man	1	0
	Herbalist - woman	1	0
	Others (in general)	0	2
Oral sources - summary	Total number	29	39
	Relatives (in total)	19	25
	Non-relatives (in total)	10	14
	Women	22	29
	Men	4	5
	Unknown sex	3	5
Non-oral sources	Books	20	10
	TV	1	3
	Magazine, newspaper	3	0
	Internet	1	2
	Radio	0	2

. Father Simon Ašič was the most frequently mentioned author; 8 informants in Karst and 5 in Gorjanci reported his books [21, 23, 24]. This finding is in accordance with a survey conducted in seven pharmacies in northeastern Slovenia [34]. From the available publications associated with medicinal plants, most of the informants (43.1%) reported reading the book *Priročnik za nabiranje rastlin* (English: Manual for wild collection of plants) by Father Simon Ašič [24]. In our study, Maria Treben was the second most reported author; two informants in Gorjanci and two in Karst reported her work *Zdravje iz božje lekarne* (English: Health through god’s pharmacy) [25]. In the previously mentioned survey, 17.8% of the informants reported reading this book [34]. Although the majority of books on medicinal plants in the Slovene language were written by pharmacists, the two most popular authors were the two with no formal pharmaceutical education.

Among the plants reported by respondents in our study, there were no plants that had not been previously reported in the popular books published in Slovenian language in 20th century. Our results therefore support the claim [88] made already in the 19th century [90] that practically all plant species found in ethnomedicinal studies in Europe are based on knowledge from old herbals. However, new and interesting details on indications, modes of preparation, and cultural and ecological influences can be found.

The influence of modern phytotherapy on folk knowledge was observed in the terminology used by the informants in the interviews. In some cases, the informants used modern terms for diseases to describe the medicinal use of reported plants, e.g., diabetes, angina pectoris, epilepsy, or depression. Moreover, in some cases, the informants reported that some plants were beneficial due to their high content of iron, minerals or vitamins. In contrast, informants frequently reported that some medicinal plants were good for the blood, weak heart, treatment of women’s problems, cleansing of blood and kidneys and other non-professional terms.

Individual people as oral sources were also important providers of botanical knowledge to the informants; 20 informants in Karst and 21 in Gorjanci reported at least one person as their teacher. It seems that women play a predominant role in the transmission of knowledge, as they were more frequently reported than men in both regions, although the sex of the source was sometimes not determined (Table 9). Relatives were more frequently reported than non-relatives (friends, co-workers, doctors, etc.), although the latter were often mentioned in both regions. Among all individual people, mothers were the most frequently reported source; eight informants in Karst and nine in Gorjanci mentioned them. Other important sources were grandmothers and friends in Karst and grandmothers, mothers-in-law and neighbors in Gorjanci.

## Conclusions

The information collected in Karst and Gorjanci and presented here provides insight into Slovenian ethnobotanical knowledge, which has received minimal study and documentation in the past. Karst and Gorjanci are two remote, rural areas where people are still connected to nature and knowledge about plants is important to their lives. There were relatively similar traditions of plant use in both areas; minor regional differences in wild-collected plants were observed, and these were mostly due to the ecological availability of the plants. The reported plants were mostly used for medicinal and nutritive purposes; it seems that it was the respondent’s intention that determined whether the plant was used as food or as medicine since both purposes were reported for approximately 50% of the plants. Numerous and diverse preparations of medicinal plants were reported for oral and topical applications. The respondents were mostly elderly people with a mean age of 61 years in Karst and 69 years in Gorjanci; however, their knowledge about plants seemed to be influenced by media, most often popular books about medicinal plants that were published in the 20th century. The results of this study may be of interest to ethnobotanists interested in the uses of plants in countries of the former Yugoslavia and for further ethnobotanical investigations in literate societies, in which folk knowledge may already be influenced by media, such as books, television and the internet.

## Abbreviations

C: Cultivated
COS: Cosmetic use
G: Gorjanci
K: Karst
MED: Medicinal use
NUT: Nutritive use
OTHER: Other uses
VET: Veterinary use
W: Wild
